# Supplementary material for: Genetic Dissection of Heat Stress Tolerance in Faba Bean (Vicia faba L.) Using GWAS
Source: Plants (Basel). 2022 Apr 20;11(9):1108. doi: 10.3390/plants11091108 (PMC9103424; doi:10.3390/plants11091108)
Supplement: Supplementary file 1 [file plants-11-01108-s001.zip › plants-1641966_supp.pdf]

Table S1: SNP associated with different traits under heat stress at Terbol summer (TRS) and Hudeiba (HD) in 2016 and 2017 seasons using ST-GWAS model.

Environment	Trait	SNP markers	allele1	allele0	MAF	Effect	P
HD16	CT	SCONTIG105792_41	G	A	0.072	1.0599	4.95E-05
HD16	CT	SCONTIG122844_17	T	G	0.173	0.6519	5.08E-05
HD16	NPP	SCONTIG123643_47	G	A	0.051	4.2376	3.86E-05
HD16	NPP	SCONTIG126455_55	T	A	0.062	4.1744	2.65E-05
**HD16**	**NSP**	**SCONTIG60075_82**	**A**	**G**	**0.133**	**2.6462**	**3.47E-06**
HD16	NPP	SCONTIG6459_80	G	A	0.1	3.1035	8.96E-05
HD16	NPP	SNODE_159317_LENGTH_67_COV_1.000000_37	C	T	0.058	4.1477	3.09E-05
**HD16**	**HSW**	**SNODE_184186_LENGTH_64_COV_1.000000_20**	**C**	**T**	**0.077**	**-19.3398**	**5.32E-05**
HD16	PLHT	SNODE_200348_LENGTH_62_COV_1.000000_39	G	A	0.267	6.3692	7.95E-05
HD16	NPP	SNODE_39759_LENGTH_70_COV_36.099998_71	G	A	0.064	4.3175	4.3E-06
HD16	CT	SNODE_4933_LENGTH_72_COV_609.527771_51	C	T	0.067	1.1366	9.31E-05
**HD16**	**GYP**	**SNODE_5492_LENGTH_64_COV_104.593750_68**	**G**	**C**	**0.379**	**1.0342**	**1.22E-05**
**HD16**	**NSP**	**SNODE_5492_LENGTH_64_COV_104.593750_68**	**G**	**C**	**0.379**	**1.8528**	**3.03E-05**
HD16	GYP	SNODE_69629_LENGTH_63_COV_1.000000_58	T	C	0.082	2.0633	7.04E-05
**HD16**	**GYP**	**SNODE_99859_LENGTH_65_COV_1.323077_30**	**C**	**T**	**0.052**	**2.9638**	**3.61E-06**
**HD16**	**NSP**	**SNODE_99859_LENGTH_65_COV_1.323077_30**	**C**	**T**	**0.052**	**5.9436**	**2.86E-07**
HD17	CT	SCONTIG50196_81	C	T	0.052	2.0946	4.18E-07
**HD17**	**GYP**	**SCONTIG60075_82**	**A**	**G**	**0.124**	**1.4752**	**2.4E-05**
**HD17**	**NPP**	**SCONTIG60075_82**	**A**	**G**	**0.124**	**1.9856**	**6.8E-07**
**HD17**	**NSP**	**SCONTIG60075_82**	**A**	**G**	**0.124**	**3.3729**	**5.38E-06**
HD17	GYP	SCONTIG92564_52	G	A	0.054	2.0239	7.2E-05
HD17	GYP	SNODE_13817_LENGTH_63_COV_1.000000_33	T	C	0.054	2.1021	3.7E-05
HD17	DFLR	SNODE_17179_LENGTH_73_COV_15.890411_64	T	G	0.179	-7.9678	4.12E-05
HD17	PLHT	SNODE_2063_LENGTH_105_COV_111.161903_87	C	T	0.362	3.2649	4.19E-05
**HD17**	**GYP**	**SNODE_39949_LENGTH_82_COV_1.024390_37**	**C**	**T**	**0.058**	**2.1881**	**5.98E-06**

**HD17**	**NPP**	**SNODE_39949_LENGTH_82_COV_1.024390_37**	**C**	**T**	**0.058**	**3.1317**	**4.19E-06**
**HD17**	**NSP**	**SNODE_39949_LENGTH_82_COV_1.024390_37**	**C**	**T**	**0.058**	**5.2417**	**8.49E-06**
HD17	DMAT	SNODE_57563_LENGTH_70_COV_5.028572_61	G	A	0.475	-3.1061	8.71E-05
TRS15	NSP	SCONTIG53986_42	C	G	0.492	-2.8965	5.02E-05
**TRS15**	**NPP**	**SNODE_2216_LENGTH_63_COV_217.412704_63**	**C**	**T**	**0.1**	**6.6171**	**1.01E-05**
TRS15	DFLR	SNODE_23772_LENGTH_65_COV_30.646154_35	G	A	0.257	5.7132	7.14E-05
TRS15	PG	SNODE_318853_LENGTH_65_COV_1.000000_65	T	A	0.105	8.6315	9.08E-06
TRS15	DFLR	SNODE_3526_LENGTH_71_COV_388.971832_48	A	G	0.07	12.7562	8.04E-05
TRS15	DFLR	SNODE_5863_LENGTH_65_COV_182.246155_71	C	T	0.129	7.9335	3.63E-05
TRS15	PG	SNODE_7398_LENGTH_62_COV_214.516129_82	G	A	0.163	6.9211	6.74E-08
TRS15	HSW	SNODE_9908_LENGTH_67_COV_43.895523_45	A	G	0.144	25.9606	3.27E-08
TRS16	NPP	SCONTIG106632_72	A	G	0.065	1.8813	6.46E-05
**TRS16**	**NPP**	**SCONTIG13629_151**	**A**	**G**	**0.186**	**0.9967**	**2.28E-05**
**TRS16**	**NSP**	**SCONTIG13629_151**	**A**	**G**	**0.188**	**1.6444**	**8.49E-05**
TRS16	NSP	SCONTIG2213_126	C	T	0.075	3.3558	3.77E-05
TRS16	NSP	SCONTIG33532_14	T	C	0.184	2.1164	6.58E-05
TRS16	NSP	SCONTIG79823_85	A	G	0.062	3.6194	1.92E-05
TRS16	GYP	SCONTIG82855_50	A	G	0.092	1.9054	1.47E-05
TRS16	NPP	SNODE_11911_LENGTH_60_COV_2.083333_21	G	A	0.065	1.9491	2.9E-05
**TRS16**	**NPP**	**SNODE_14795_LENGTH_67_COV_68.791046_60**	**T**	**C**	**0.115**	**1.2877**	**2.43E-06**
**TRS16**	**NSP**	**SNODE_14795_LENGTH_67_COV_68.791046_60**	**T**	**C**	**0.116**	**2.3434**	**1.12E-06**
TRS16	PG	SNODE_23446_LENGTH_112_COV_17.633928_126	G	A	0.455	5.6823	4.5E-05
TRS16	NSP	SNODE_344_LENGTH_74_COV_134.351349_70	A	G	0.054	3.4896	9.4E-05
TRS16	PG	SNODE_40068_LENGTH_63_COV_13.873015_43	A	G	0.383	4.0078	5.22E-05
TRS16	DMAT	SNODE_49877_LENGTH_67_COV_2.925373_66	C	T	0.095	4.6696	6.66E-06
TRS16	PG	SNODE_6662_LENGTH_69_COV_474.000000_31	T	C	0.156	5.3462	8.73E-05
**TRS16**	**NPP**	**SNODE_6820_LENGTH_63_COV_471.317474_68**	**T**	**A**	**0.052**	**2.2451**	**6.96E-05**
**TRS16**	**NSP**	**SNODE_6820_LENGTH_63_COV_471.317474_68**	**T**	**A**	**0.053**	**4.0527**	**4.6E-05**
TRS16	DFLR	SNODE_7114_LENGTH_70_COV_20.785715_58	C	T	0.11	7.2768	7.47E-07

**TRS16**	**NPP**	**SNODE_7399_LENGTH_66_COV_159.287872_84**	**C**	**T**	**0.106**	**1.1522**	**6.4E-05**
**TRS16**	**NSP**	**SNODE_7399_LENGTH_66_COV_159.287872_84**	**C**	**T**	**0.107**	**2.2333**	**1.01E-05**
TRS16	PG	SNODE_7979_LENGTH_116_COV_512.344849_106	A	G	0.078	10.1607	9.27E-05
TRS17	GYP	SCONTIG10268_58	A	G	0.069	9.2527	5.48E-05
TRS17	GYP	SCONTIG127798_64	G	T	0.063	7.9077	1.69E-05
TRS17	PLHT	SCONTIG30232_88	C	G	0.36	-7.6776	2.26E-05
**TRS17**	**NPP**	**SCONTIG82391_71**	**T**	**G**	**0.093**	**15.522**	**4.71E-05**
**TRS17**	**NSP**	**SCONTIG82391_71**	**T**	**G**	**0.093**	**27.0305**	**2.14E-06**
TRS17	GYP	SNODE_1409_LENGTH_91_COV_549.780212_63	G	T	0.076	7.9499	4.02E-06
**TRS17**	**GYP**	**SNODE_2216_LENGTH_63_COV_217.412704_63**	**C**	**T**	**0.1**	**9.2485**	**5.88E-06**
TRS17	PG	SNODE_38942_LENGTH_69_COV_118.768112_43	A	G	0.076	7.7743	7.95E-05
TRS17	PLHT	SNODE_41542_LENGTH_59_COV_3.338983_65	C	G	0.35	-7.899	5.15E-06
TRS17	GYP	SNODE_59818_LENGTH_86_COV_1.104651_64	G	T	0.089	6.6134	3.31E-05
TRS17	PLHT	SNODE_936_LENGTH_67_COV_546.776123_65	C	G	0.283	-7.5774	2.51E-05

HD16: Hudeiba 2015/2016; HD17: Hudeiba 2015/2016; TRS15: Terbol summer planting 2015; TRS16: Terbol Summer planting 2016; TRS17: Terbol summer planting 2017

Table S2: Multi-trait genome-wide association study (MT-GWAS) results for grain yield and yield components. Underlined p-values are the significant p-values while underlined SNPs are the SNPs that were detected in multiple traits. AF: allele frequency of allele1

Trait	QTLID	SNP	allele1	allele0	AF	P
NPP	NPP_1	SCONTIG124089_41	T	G	0.05	3.10E-05
	NPP_2	SCONTIG42136_36	C	T	0.2	3.90E-05
	NPP_3	SCONTIG57861_63	G	A	0.12	3.40E-05
	NPP_4	SCONTIG60075_82	A	G	0.15	4.60E-05
	NPP_5	SCONTIG97070_42	T	C	0.06	3.20E-05
	NPP_6	SNODE_19219_LENGTH_71_COV_2.605634_17	G	A	0.08	2.00E-05
	NPP_7	SNODE_39949_LENGTH_82_COV_1.024390_37	C	T	0.06	4.80E-05

	**NPP_8**	**SNODE_40333_LENGTH_77_COV_34.987015_87**	**T**	**A**	**0.18**	**3.70E-06**
	**NPP_9**	**SNODE_559376_LENGTH_95_COV_1.252632_60**	**A**	**T**	**0.45**	**6.20E-12**
	NPP_10	SNODE_5667_LENGTH_63_COV_224.857147_29	G	A	0.06	8.30E-05
	NSP_1	SCONTIG10268_47	C	A	0.06	4.40E-05
	NSP_2	SCONTIG124089_41	T	G	0.05	2.60E-05
	NSP_3	SCONTIG60075_82	A	G	0.14	4.40E-05
	**NSP_4**	**SCONTIG82391_71**	**T**	**G**	**0.09**	**3.50E-06**
	**NSP_4**	**SCONTIG82391_72**	**C**	**T**	**0.09**	**3.50E-06**
NSP	**NSP_4**	**SCONTIG82391_73**	**A**	**T**	**0.09**	**3.50E-06**
	**NSP_5**	**SNODE_11884_LENGTH_82_COV_596.182922_61**	**A**	**G**	**0.09**	**1.00E-07**
	NSP_6	SNODE_159732_LENGTH_67_COV_1.000000_17	A	G	0.48	4.40E-05
	NSP_7	SNODE_18243_LENGTH_66_COV_1.000000_69	G	A	0.44	9.50E-06
	NSP_8	SNODE_39949_LENGTH_82_COV_1.024390_37	C	T	0.06	4.50E-05
	NSP_9	SNODE_47942_LENGTH_114_COV_3.815789_60	A	G	0.08	8.90E-05
	HSW_1	SCONTIG124448_54	A	C	0.1	4.30E-05
	HSW_2	SCONTIG16540_60	C	T	0.08	3.50E-05
	HSW_3	SNODE_12987_LENGTH_81_COV_566.222229_77	A	C	0.15	1.60E-05
HSW	HSW_4	SNODE_18703_LENGTH_72_COV_258.527771_54	T	C	0.49	4.20E-05
	HSW_5	SNODE_8714_LENGTH_71_COV_9.901408_38	A	G	0.38	2.40E-05
	**HSW_6**	**SNODE_9908_LENGTH_67_COV_43.895523_45**	**A**	**G**	**0.17**	**7.10E-07**
	HSW_7	SNODE_99859_LENGTH_65_COV_1.323077_16	C	T	0.07	5.00E-05
	GYP_1	SCONTIG2213_126	C	T	0.07	4.80E-05
	GYP_2	SCONTIG60075_82	A	G	0.13	1.60E-05
GYP)	**GYP_3**	**SCONTIG72702_49**	**A**	**G**	**0.5**	**2.30E-06**
	GYP_4	SNODE_14795_LENGTH_67_COV_68.791046_60	T	C	0.08	4.70E-05

	GYP_5	SNODE_39949_LENGTH_82_COV_1.024390_37	C	T	0.05	4.80E-05
	GYP_6	SNODE_40333_LENGTH_77_COV_34.987015_87	T	A	0.17	8.60E-06
	GYP_7	SNODE_5492_LENGTH_64_COV_104.593750_68	G	C	0.38	3.30E-05
DFLR	DFLR_1	SNODE_300878_LENGTH_65_COV_1.000000_26	G	A	0.07	3.00E-05
	DFLR_2	SNODE_4853_LENGTH_74_COV_297.162170_75	C	T	0.12	7.80E-05
DMAT	DMAT_1	SNODE_10083_LENGTH_100_COV_16.170000_92	T	C	0.11	1.60E-05
PLHT	PLHT_1	SNODE_126238_LENGTH_69_COV_1.000000_21	T	C	0.51	9.90E-06
	PLHT_1	SNODE_126238_LENGTH_69_COV_1.000000_60	C	T	0.51	9.90E-06
	PLHT_2	SNODE_144193_LENGTH_65_COV_1.000000_69	C	A	0.46	4.10E-05
	PLHT_3	SNODE_150825_LENGTH_64_COV_1.000000_31	C	A	0.06	8.40E-06
	PLHT_3	SNODE_150825_LENGTH_64_COV_1.000000_42	A	C	0.06	8.40E-06
	PLHT_3	SNODE_150825_LENGTH_64_COV_1.000000_64	C	T	0.06	8.40E-06
	PLHT_4	SNODE_88919_LENGTH_65_COV_1.000000_26	A	G	0.14	6.00E-06
CL (%)	CHLC_1	SNODE_11261_LENGTH_62_COV_1.048387_42	C	T	0.32	8.10E-05
CT	CT_1	SCONTIG122844_17	T	G	0.17	9.70E-05
	**CT_2**	**SCONTIG50196_81**	**C**	**T**	**0.06**	**6.00E-07**
PG (%)	**PG_1**	**SCONTIG82855_50**	**A**	**G**	**0.09**	**1.50E-06**
	PG_2	SNODE_318853_LENGTH_65_COV_1.000000_65	T	A	0.11	6.50E-05
	PG_3	SNODE_38942_LENGTH_69_COV_118.768112_43	A	G	0.07	3.10E-05
	PG_4	SNODE_50590_LENGTH_70_COV_3.571429_27	G	C	0.33	5.70E-05
	PG_5	SNODE_6662_LENGTH_69_COV_474.000000_31	T	C	0.16	1.30E-05
	PG_6	SNODE_7398_LENGTH_62_COV_214.516129_66	C	T	0.16	1.30E-05
	**PG_6**	**SNODE_7398_LENGTH_62_COV_214.516129_82**	**G**	**A**	**0.16**	**3.50E-07**
	PG_7	SNODE_7979_LENGTH_116_COV_512.344849_106	A	G	0.08	1.60E-05
